# Sustainable Value Co-Creation in the Virtual Community: How Diversified Co-Creation Experience Affects Co-Creation Intention

**DOI:** 10.3390/ijerph17228497

**Published:** 2020-11-17

**Authors:** Yi-Wen Chen

**Affiliations:** Department of Information and Communication, Tamkang University, New Taipei City 237, Taiwan; 137186@mail.tku.edu.tw

**Keywords:** value co-creation, co-creation experience, theory of planned behavior, online game

## Abstract

The popularization of digital infrastructure has enabled the rise of the online game industry. Instead of targeting entertainment-oriented technology and services, which are the focus of most relevant studies, in the present study, we review the literature from the perspective of considering players of online games as both consumers of entertainment and co-creators of value. The three major antecedents of the theory of planned behavior, namely personal attitude toward co-creation, subjective norms and perceived behavioral control, were modified to explore the relevant constructs. Specifically, the diversity of co-creation experience was used to predict co-creation intention. The proposed model was empirically evaluated through the structural equation modeling of survey data collected from 321 World of Warcraft (WoW) players. As hypothesized, the diversified co-creation experience positively affected the antecedents. The findings provide implications on how to increase players’ participation in co-creation to achieve sustainable mutual benefits.

## 1. Introduction

Since the inception of the concept of co-creation, scholars and practitioners have paid increasing attention to company–customer interactions [1]. According to the conventional concept in marketing or strategy research, value is generated by a firm through its value chain. From the customer’s perspective, co-creation experience can create unique value [2]. Because consumers play a crucial role in developing co-creation value, studies on service-dominant (S-D) logic are increasing [3,4,5,6,7,8,9,10]. Its core concepts includes the effects of customer participation [11,12], customer behavior [7,13] and measurement and scales [5,7,14,15,16]. Not only do firms benefit considerably from customer feedback; after improvements are made accordingly, customers receive better service. Because the generation of mutual value enables the formulation of stable, long-term business plans, the issue of sustainability has also received scholarly attention [17,18]. Furthermore, several studies have pointed out the strong relationship between sustainability, open innovation and value co-creation [9,10].

Sustainable value co-creation is applied in information infrastructure development around the globe. During the past decade, there has been a rise of application platforms that provide accessible virtual environments that allow user involvement in co-production processes. For instance, users of social media platforms such as blogs, YouTube or Facebook not only view but create content. These online communities permit faster, richer and more frequent interactions among large groups of people. Their success has been driven by the fact that, in the digital age, most users are proficient with computers, information and multimedia technology in general. As digital trends reshape customer behaviors, scholarly interest in customer engagement in virtual environments has increased [19,20,21,22,23]. The success of creating co-creation value depends on a large number of experienced users participating in co-creation activities. In the video game industry, a previous study found that WoW players increase their abilities by earning experience points by completing different tasks [24]. Thus, the diversified co-creation experience is a critical factor in exploring users’ co-creation intention.

Typically, social influence and social networks are determinants of customer co-creation intention and behavior, especially in the virtual community [22,25,26,27]. However, interpretations of the meaning and scope of co-creation vary. From the social aspect of consumer behavior, studies have proposed several explanations for the phenomenon in which two or more participants engage in collective action: we-intention [28,29,30,31], collective intention [32,33] and co-creation intention [34,35]. Although studies have applied and extended the theory of planned behavior (TPB) to explain and predict customer co-creation in social media or commerce contexts, they have only identified social factors as key drivers of participation (i.e., involvement or social support), rather than holistically examining antecedents of co-creation from the social influence perspective [35,36].

Perceived behavioral control is a critical variable of the TPB.; however, a previous meta-analysis indicated that the concept of self-efficacy is defined more clearly and is more strongly correlated with intention [37]. Because self-efficacy is generated from prior experience and challenging tasks [38], especially in the computer domain [39], co-creation experience is relevant to its discussion [40,41,42]. Thus, the present study applies the TPB framework and modifies the antecedents of co-creation intention in the context of the virtual community.

The purpose of this study is mainly to investigate the relationships between diversified experiences of co-creation and the three core elements of the TPB model in the context of a virtual community. This research makes two theoretical contributions: first, it extends the TPB model with diversified co-creation experience; second, the study demonstrates the modification of constructs in the context of virtual communities. Specifically, this study employs the social influence perspective to develop the conceptual model. The organization of the article is as follows: first, this paper explores the relevant literature on diversified experience and identifies three antecedents of the TPB.; next, the hypothesis development and the research framework are proposed; third, nine hypotheses are examined by using a structural equations model; finally, a discussion and conclusions are provided to present the findings and implications and suggest directions for future research.

## 2. Literature Review and Hypothesis Development

The TPB model has been extensively used to predict individual intention since Ajzen proposed an extension of the theory of reasoned action [43], and it has been validated and extended in numerous fields. Its core elements comprise personal attitude toward intention (or a specific behavior), subjective norms (i.e., the perception of social norms) and the perception of behavioral control (i.e., the feeling of having control over behavior) [43,44]. The TPB model predicts different types of intentions. To explain co-creation intention, its elements have been modified to include conditions such as participation [45], involvement [35] and past co-creation behavior [46]. These interactive experiences enhance one’s ability to create “knowledge corridors” and discover opportunities [47,48,49], potentially exerting a strong impact on one’s decisions and behavior. Thus, experience is the primary core value of co-creation activities [2,40]; co-creation experience contributes substantially to the explanatory richness of the TPB model [35,50,51].

### 2.1. Diversified Experience of Co-Creation and Personal Attitude

Experience can be viewed as one’s observation of environments [52]. Real-world experiences affect individuals’ perception and expression of values regarding a specific role [53], which are the most important foundational factors affecting personal attitudes. Several studies have confirmed the relationship between experience and attitudes [54,55,56,57]. In the online game context, user experience positively affects four values (i.e., information and experiential, social and transaction value) that determine user attitudes [58]. In the case of social media, the users’ depth of engagement in communication determines their community commitment, which in turn positively affects personal attitudes [59]. Most prior studies have pointed out only that the experience is an important antecedent affecting core elements of the TPB model [60,61]; the effects of experience types or levels on personal attitude have not been investigated in previous research. In the context of a virtual community, when users trust that participating in different tasks will broaden their understanding of the co-creation activities of interest, they will have a more favorable attitude toward co-creation.

Specifically, the experience-scape likely produces favorable attitudes [62]. Because personal attitudes are shaped by values and commitment, having a diverse range of experiences facilitates the development of self-confidence [63], which results in higher levels of personal attitude [64]. Given these considerations, the following hypothesis is proposed:

**Hypothesis** **1** **(H1).** *A diversified experience of co-creation is positively related to attitude toward co-creation intention.*

### 2.2. Diversified Experience of Co-Creation and External Subjective Norms

Subjective norms refer to the perceived social pressure from significant others such as family, friends and colleagues, whose approval of one’s decisions is important [65]. The concept was originally defined in the TPB model [43] as a social factor that reflects the perception of reference people [65] or cohesive referents [66]. Studies conducted on the basis of this model have reported a positive effect of experience on subjective norms [60,67]. Studies of technology acceptance suggest that social influencing factors, particularly subjective norms, should consider the influence of external referents on the strong social relationships and connections in the virtual community [68]. The magnitude of the influence of one’s perception by opinion leaders and key community members is significantly higher than that of real-world significant others. Considering this, one study extended the concept of referents in digital relationships to include external referents and proposed external subjective norms as a factor affecting these connections [68].

Little work has been conducted to examine the relationship between experience and subjective norms. Most previous researchers have not explored the relationships between two variables that influence intention [69,70], while only a few studies have focused on examining the effect of flow experience on subjective norms [70]. Social media users with co-creation experience linked to practical problem-solving activities typically perceive greater social support than newcomers from the virtual community [71]. Because participants are socially influenced by their peers and referents, social (including informational and emotional) support from community members significantly affects subjective norms [72]. Experiences in online communities also positively affect social identity [73]—a predictor of external subjective norms [68]. On the basis of this discussion, the following hypothesis is proposed:

**Hypothesis** **2** **(H2).** *A diversified experience of co-creation is positively related to external subjective norms.*

### 2.3. Diversified Experience of Co-Creation and Creative Self-Efficacy

Perceived behavioral control, defined in the TPB model as the self-evaluation of the ease or difficulty with which behaviors are performed [43], is similar to self-efficacy [60,74] and strongly predicts behaviors of interest [75]. Specifically, co-creation is a process in which a consumer actively engages in the production of creative outcomes [76,77]. Thus, creative self-efficacy—the belief that one has the ability to produce creative outcomes [78]—should be considered in the creativity context [78,79]. Several studies suggest that experience is crucial for shaping creative self-efficacy [80,81]; however, other researchers found conflicting results [82]. However, those results are generated by using a training period to measure experience. In the context of a virtual community, users can participate in different co-creation activities which provide them with a broad range of experience.

Diversity has been discussed as a major driver of creativity [83]. Most relevant studies have focused on team diversity [84,85], cognitive diversity [86] and knowledge diversity [87,88]. Diversity may generate benefits for creative performance, because varied knowledge and information richness help people to “think outside the box”. Entrepreneurship studies that embrace the “jack-of-all-trades’’ view [89] have indicated that experiential diversity increases the possibilities of becoming an entrepreneur [90,91,92]. Entrepreneurial abilities, skill and knowledge are developed through learning by doing [93]. According to one study, the existence of and exposure to external knowledge increase self-confidence, and varied knowledge can enhance self-efficacy [94]. Engaging in co-creation, which involves performing varied tasks, in turn increases co-creation abilities, positively affecting users’ self-judgment of their creative contribution. Considering these findings, the following hypothesis is proposed:

**Hypothesis** **3** **(H3).** *A diversified experience of co-creation is positively related to creative self-efficacy.*

### 2.4. Diversified Experience of Co-Creation and Co-Creation Intention

Co-creation experiences—the core concept of value co-creation [2,40,41,42]—are generated from co-creation activities; therefore, personalized interactions are crucial for creating value [2]. Virtual community studies have indicated that co-creation experiences have a positive effect on the intention of future participation [77,95]. Furthermore, the co-creation experience is a vital predictor of purchase intention [96].

Co-creation experiences, which are classified into learning value, social integrative value and hedonic value, are driven by better customer experiences and evaluations [77]. Therefore, this study presents the following hypothesis:

**Hypothesis** **4** **(H4).** *A diversified experience of co-creation is positively related to co-creation intention.*

### 2.5. Components of TPB Model

The three elements of the TPB model, namely personal attitude toward co-creation, subjective norms and perceived behavioral control, all have positive impacts on co-creation intention [35,46]. When an individual first develops a favorable attitude toward co-creation, the intention of co-creation will become stronger [35]. Because co-creation participation is considered in the virtual community context, social perspectives must be considered to explain the influence of significant others [69], such as key opinion leaders or celebrities, on decision-making. According to a study on customers’ willingness to participate in value co-creation in a virtual brand community, users expect their knowledge contribution to be equal to or higher than the cost of their investment [95]. Based on these perspectives, the following hypotheses are proposed:

**Hypothesis** **5** **(H5).** *Personal attitude is positively related to co-creation intention.*

**Hypothesis** **6** **(H6).** *External subjective norms are positively related to co-creation intention.*

**Hypothesis** **7** **(H7).** *Creative self-efficacy is positively related to co-creation intention.*

In the original version of the TPB, subjective norms are one of the motivational factors that have a direct impact on intention [43]. Furthermore, the relationships between subjective norms and both personal attitude and perceived behavioral control have not been further explored. The empirical evidence indicates that the construct of subjective norms positively influences both personal attitude and self-efficacy [60]. Thus, this study proposes the following hypotheses:

**Hypothesis** **8** **(H8).** *External subjective norms are positively related to personal attitude.*

**Hypothesis** **9** **(H9).** *External subjective norms are positively related to creative self-efficacy.*

### 2.6. Research Framework

**Figure 1:**
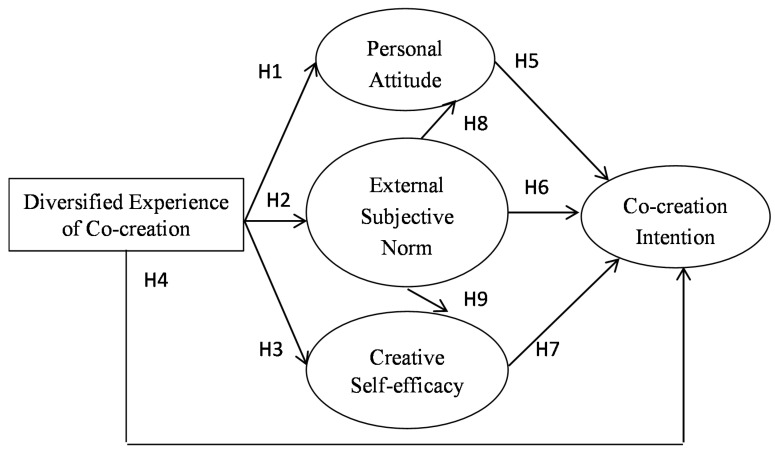
Research framework.

The TPB model has been widely applied to the examination of specific behaviors; however, few studies have explored the relationship between diversified experiences and co-creation in a virtual community. The purpose of this study is to extend the TPB model by modifying its three core elements and by integrating the social perspective with the creative perspective. A diversified experience was used to explain the influencing factors of the model because diversity is the root of creativity. Moreover, external subjective norms were considered to highlight the importance of social contexts because key opinion leaders exert considerable influence in the virtual community. Compared with other behaviors, co-creation relies more strongly on creative abilities. Therefore, the present study emphasized the self-evaluation of knowledge and capability through the concept of creative self-efficacy. Figure 1 shows the research framework, encompassing evidence from the TPB model, social theory and the creative perspective.

## 3. Methodology

### 3.1. Data Collection and Sampling

Data were collected through an online questionnaire survey of active players of WoW in Taiwan, which yielded 321 usable responses. Data collection took place in Bahamut (http://www.gamer.com.tw), which is the biggest online community of AGC (anime, comics and games) in Taiwan. According to the information from the Bahamut website, the membership of this community is over 2.5 million members. WoW was released in 2004 by Blizzard Entertainment and is a massively multiplayer online role-playing game (MMORPG). The MMORPG game exhibits a specific characteristic of user-generated content (UGC), and WoW is one of the most successful co-creation platforms in online game industries. The questionnaire used in this research was posted on the forum of WoW. The message stated the purpose of this study abd displayed a hyperlink to the survey form on mysurvey (http://www.mysurvey.tw/). In order to improve the valid response rate, reliability and validity of the questionnaire, gifts were provided to respondents. The descriptive statistics of the variables (i.e., gender, age, education and years of online game and co-creation experience) are presented in Table 1.

### 3.2. Measurements

The items used were based on those in the literature. According to Bagozzi and Dholakia [29], user engagement in Linux open-source communities can be divided into five types, including participating in joint interactions, visiting websites, and working with the company. Thus, diversified experience and co-creation intention in a specific field should be classified by the level of user participation. In the case of WoW, four co-creation activities that can generate value for both the firm and users were determined through an interview consultation with six experts. Those experts were invited from Bahamut (http://www.gamer.com.tw). The first step was to post the requirements of this study on the forum of WoW, and the six respondents, who had played WoW for over 10 years, were selected for an interview. The second step was to conclude all the co-creation activities mentioned by each expert. The four activities of co-creation, namely participating in discussion, solving and debugging problems, creating macro codes and coding and developing plug-ins, were all confirmed in discussion. Specifically, diversified experience was measured as a player’s sum of these activities, ranging from 0 to 4.

The remaining constructs were scored on a seven-point Likert scale, from 1 (strongly disagree) to 7 (strongly agree). The scales for personal attitude and external subjective norms were adapted from studies by Ajzen [43] and Song and Kim [68] and comprised three and two items, respectively. Creative self-efficacy and co-creation intention, both measured using three items, were based on studies by Tierney and Farmer [78] and Lee [70], respectively. Because four types of co-creation intention were considered, each item of co-creation intention was measured independently. The measurement items are listed in Table A1 in Appendix A.

## 4. Empirical Results

Structural equation modeling (SEM) was used for hypothesis verification, and the results were obtained using maximum likelihood estimation and AMOS (version 18.0) software (IBM, Armonk, NY, US).

### 4.1. Measurement Model Results

Table 2 shows the means and standard deviations of the constructs among which significant correlations were observed, except for those between diversified experience of co-creation and external subjective norms.

The fitness measures were all within an acceptable range; the indicators included the goodness of fit (GFI) = 0.936, comparative fit index (CFI) = 0.968, normed fit index (NFI) = 0.953 and root mean square error of approximation (RMSEA) = 0.078. Table 3 lists detailed information regarding the reliability and convergent and discriminant validity. The diversified experience of co-creation was the sole construct measured using an observed item. Loadings of each construct were evaluated. All values of Cronbach α values and average variance extracted (AVE) exceeded the acceptable values of 0.7 and 0.5, indicating satisfactory reliability and convergent validity, respectively. Furthermore, the square root AVE of any two constructs exceeded the constructs’ correlation coefficient (Table 2), indicating adequate discriminant validity.

### 4.2. Structural Model Testing

**Figure 2:**
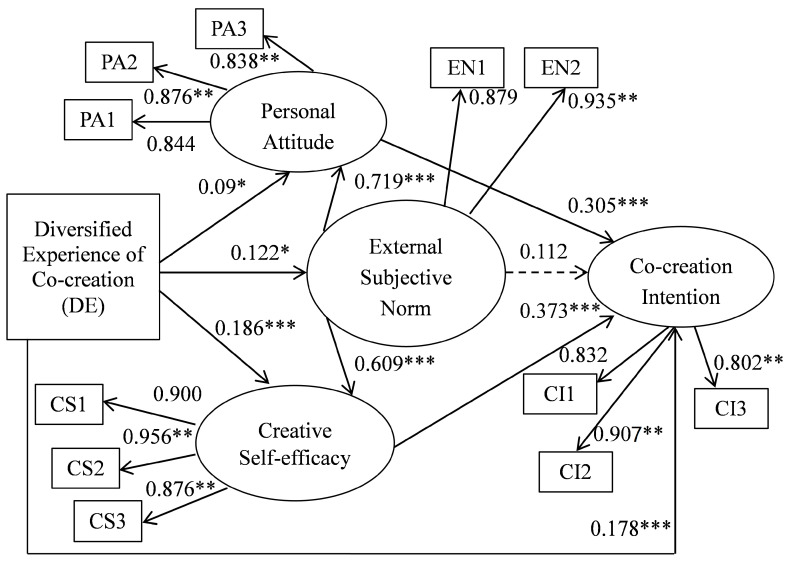
The result of the full model. Note: * *p* < 0.05, ** *p* < 0.01, *** *p* < 0.001.

The structural model was tested using AMOS 18. Figure 2 shows the results, including the standardized path coefficients between constructs, with dotted lines denoting non-significant paths. The fit indices demonstrated that the model fit the data well (χ^2^ = 139.024, degrees of freedom (d.f.) = 47, χ^2^/d.f. = 2.958, GFI = 0. 936, IFI = 0. 968, CFI = 0. 968, NFI = 0.953, RMSEA = 0.078). Table 4 presents the hypothesized paths. All hypotheses, except hypothesis 6, were supported by the model.

## 5. Discussion

As mentioned, co-creation behavior in online game communities was explained using SEM to test and extend the TPB model. The results demonstrated that a diversified experience of co-creation contributes critically to the three major components of the TPB model. Specifically, experience of participation in different tasks improved players’ attitudes, external subjective norms and creative self-efficacy. The effects of a diversified experience on three effects were statistically significant, as shown by the path coefficients of 0.09 (*p* < 0.05), 0.122 (*p* < 0.05) and 0.186 (*p* < 0.001). Therefore, hypotheses 1, 2 and 3 were supported. This outcome shows that a diversified experience has the strongest impact on creative self-efficacy. Moreover, this diversified experience positively affects co-creation intention. As the path coefficient was 0.178 (*p* < 0.001), hypothesis 4 was supported. These findings are consistent with those of previous studies that noted that past experiences have significant effects on antecedents of intention [60,97]. In addition, a diversified experience motivates individuals to engage in co-creation; this extends the original TPB model. Notably, the influence of a diversified experience on creative self-efficacy was much stronger than that of the other constructs. A possible explanation for this is that participation in varied co-creation activities facilitates the development of various skills, strengthening players’ confidence in their capacity for creative contribution.

Studies have confirmed that the three core elements of the TPB (i.e., attitude toward co-creation, external subjective norms and perceived behavioral control) predict co-creation intention in online games [35,46]. Consistent with these expectations, the path coefficients from personal attitude and creative self-efficacy to co-creation intention were statistically significant, as shown by the path coefficients of 0.305 (*p* < 0.001) and 0.373 (*p* < 0.001). As the two antecedents exerted a very strong impact on intention, hypotheses 5 and 7 were supported. However, in the present study, external subjective norms did not positively affect intention but had an indirect effect. According to the statistical result, hypothesis 6 was not supported. The positive effects of external subjective norms on players’ attitudes and creative self-efficacy were very strong, as indicated by the path coefficients of 0.719 (*p* < 0.001) and 0.609 (*p* < 0.001). Consequently, hypotheses 8 and 9 were supported. This is in line with the findings of Liñán and Chen [60], who claimed that social pressures affect levels of attitude and perceived behavioral control. They also indicated that people hold more favorable attitudes toward and are more confident regarding their ability to perform specific behaviors when they have their significant others’ approval [60].

## 6. Limitations, Implications and Future Research

This study has some limitations. The measurement of the diversified experience of co-creation in one particular online game and the use of a single study setting may limit the generalizability of the findings. Future research should validate the proposed model through the use of different games and include larger samples from different countries. In addition, because a cross-sectional design was used, causal inferences could not be made. Therefore, future longitudinal studies are warranted.

## 7. Conclusions

This section is mandatory. Please summarize the main achievements and/or results in this section. This study has extended the TPB model. The results show that the diversified co-creation experience is a crucial determinant in predicting co-creation intention in online games. Four implications for practitioners concerning the development of a sustainable co-creation platform in the online game industry can be drawn from the present findings. First, online game managers should be aware of the importance of diversified experiences of co-creation, and more diversified tasks should be designed to encourage more players to participate and thereby improve their attitude toward engaging in such activities. This can also improve their perceptions of external subjective norms, which can in turn positively affect creative self-efficacy—which also influences intention. The greatest benefit is increasing players’ self-confidence; companies can gain from better-trained players who can contribute their knowledge and skill to co-creation. Second, companies can develop an attractive reward system with diversified tasks to enrich players’ co-creation experience. Notably, because key opinion leaders or experts in the virtual community shape users’ decisions to participate in co-creation, their comments or advice should be included in the program. Third, because creative self-efficacy is the strongest predictor of co-creation intention of all the antecedents of the TPB model, companies should develop a range of co-creation activities that vary in difficulty to accommodate players of all levels and enable them to hone their abilities incrementally. Finally, managers should encourage peers or top influencers to shape collaborative culture in online communities. For instance, online game companies can organize or support some online/offline gatherings for co-creators.

## Figures and Tables

**Table 1:** Profile of respondents.

Measure	Frequency	Percentage (%)
Gender		
Male	244	76
Female	77	24
Age (years)		
≤20	52	16.2
21–25	134	41.7
26–30	77	24
31–35	38	11.8
36–40	15	4.7
41–50	4	1.2
≥51	1	0.3
Education		
Junior high school (or below)	8	2.5
General/Vocational high school	56	17.4
College/University	216	67.3
Bachelor’s degree (or above)	41	12.8
Years of playing on-line game experiences		
<1	92	28.7
1–2	64	19.9
3–4	75	23.4
5–6	50	15.6
≥7	40	12.5
Years of engaging co-creation activities experiences		
<1	200	62.63
1–2	61	19
3–4	34	10.6
5–6	15	4.7
≥7	11	3.4

**Table 2:** Means, standard deviations and correlations of the constructs.

Constructs	Mean	Standard Deviation	A	B	C	D	E
A. Diversified experience of co-creation	1.514	0.802					
B. Personal attitude	5.039	1.095	0.170 **				
C. External subjective norm	4.841	1.155	0.103	0.641 **			
D. Creative self-efficacy	4.868	1.252	0.267 **	0.578 **	0.576 **		
E. Co-creation intention	4.800	0.906	0.342 **	0.508 **	0.465 **	0.600 **	

Note: ** *p* < 0.01.

**Table 3:** Analysis of reliability and convergent and discriminant validity of the measurement model. AVE: average variance extracted.

Constructs	Items	λ	Cronbach’s α	AVE	The Square Root of AVE
A. Diversified experience of co-creation	DE	The observed variable			
B. Personal attitude	PA1 PA2 PA3	0.844 0.876 ** 0.838 **	0.887	0.727	0.852
C. External subjective norm	EN1 EN2	0.879 0.935 **	0.905	0.823	0.907
D. Creative self-efficacy	CS1 CS2 CS3	0.900 0.956 ** 0.876 **	0.933	0.830	0.911
E. Co-creation intention	CI1 CI2 CI3	0.832 0.907 ** 0.802 **	0.880	0.719	0.847

Note: ** *p* < 0.01.

**Table 4:** Path coefficient analysis.

Hypothesis	Results	Path Coefficient
H1	H1 is supported	0.09 *
H2	H2 is supported	0.122 *
H3	H3 is supported	0.186 ***
H4	H4 is supported	0.178 ***
H5	H5 is supported	0.305 ***
H6	H6 is not supported	0.112
H7	H7 is supported	0.373 ***
H8	H8 is supported	0.719 ***
H9	H9 is supported	0.609 ***

Note:* *p* < 0.05, ** *p* < 0.01, *** *p* < 0.001.

## Appendix A

**Table A1:** Questionnaire items.

Constructs	Numbers	Items	References
A. Diversified experience of co-creation	DE	Please check the following co-creation activity that you have been participated when playing an on-line game.	Interview
		0 = never; 1 = participating in discussion; 2 = solving and debugging problems; 3 = creating a macro code; 4 = coding a developing plug-ins	
B. Personal attitude	PA1	I feel good about participating in co-creation activity when playing an online game.	Ajzen [43]
	PA2	I like participating in co-creation activity when playing an online game.	
	PA3	I think that participating in co-creation activity when playing an online game is a good leisure activity.	
C. External subjective norm	EN1	Experts whose comments I rely on in an online game community have provided supporting evidence for participating in co-creation activity.	Song & Kim [68]
	EN2	My friends whose opinions I think important in an online game community have provided supporting evidence for participating in co-creation activity.	
D. Creative self-efficacy	CS1	I have confidence in my ability to solve problems creatively when participating in co-creation activity in an online game.	Tierney & Farmer [78]
	CS2	I feel that I am good at generating novel ideas when participating in co-creation activity in an online game.	
	CS3	I have a knack for further developing the ideas of others when participating in co-creation activity in an online game.	
E. Co-creation intention	CI1	Average of CI1_1 to CI1_4	Lee [70]
	CI1_1	It’s worth participating in discussion when playing online games.	
	CI1_2	It’s worth solving and debugging problems when playing online games.	
	CI1_3	It’s worth creating a macro code when playing online games.	
	CI1_4	It’s worth developing plug-ins when playing online games.	
	CI2	Average of CI2_1 to CI2_4	
	CI2_1	I will frequently participate in discussion when playing an online game.	
	CI2_2	I will frequently solve and debug problems when playing an online game.	
	CI2_3	I will frequently create a Macro code when playing an online game.	
	CI2_4	I will frequently develop plug-ins when playing an online game.	
	CI3	Average of CI3_1 to CI3_4	
	CI3_1	I would be willing to recommend participainge in discussion when playing an online game to other people.	
	CI3_2	I would be willing to recommend solving and debugging problems when playing an online game other people.	
	CI3_3	I would be willing to recommend creating a macro code when playing an online game to other people.	
	CI3_4	I would be willing to recommend developing plug-ins when playing an online game to other people.	
